# Increasing Trends of Artificial Intelligence With Robotic Process Automation in Health Care: A Narrative Review

**DOI:** 10.7759/cureus.69680

**Published:** 2024-09-18

**Authors:** Prashant Nimkar, Deepika Kanyal, Shantanu R Sabale

**Affiliations:** 1 Hospital Administration, Datta Meghe Institute of Higher Education and Research, Wardha, IND; 2 Hospital Administration, Datta Meghe Institute of Medical Sciences, Wardha, IND; 3 Hospital Administration, Jawaharlal Nehru Medical College, Wardha, IND

**Keywords:** appointment scheduling, electronic health records, health care efficiency, machine learning, remote patient monitoring, telehealth

## Abstract

This review explores the fast-growing importance of artificial intelligence (AI) with robotic process automation (RPA) in healthcare. AI uses intelligent algorithms to analyze data, while RPA automates repetitive tasks to improve efficiency and accuracy. These technologies are swiftly revolutionizing health care by improving diagnostic precision, accelerating administrative tasks, reducing operation timing, and improving patient care. Application of these technologies requires good technical understanding, preparedness for continuous learning, and adaptability to new challenges. This review aims to provide an in-depth study of the potential applications, present implementations, challenges, and future scope of AI with RPA in healthcare. It can provide information to researchers, professionals, and decision-makers regarding the application of the technologies under consideration for better productivity, increased security and accuracy of data, cost reduction, and personalization of healthcare provided to patients. The main results are that AI and RPA can ensure greater data security, provide supporting work in administration, like scheduling appointments and medical billing, make better decisions, enable telehealth and remote patient monitoring, reduce human error, and increase overall health outcomes. This review overviews the challenges in implementing robotics technology, focusing mainly on secondary source journals, scholarly articles, and reference books. Key findings indicate that this study reveals how robotics could alleviate healthcare professionals. Further research, investment, and collaboration will be needed to enable these technologies to reach their full potential for healthcare delivery. However, challenges such as data privacy and security concerns, high implementation costs, and regulatory and ethical considerations must be addressed. The conclusion emphasizes that while these technologies are revolutionizing healthcare by increasing efficiency and personalizing patient care, ongoing research, investment, and collaboration are essential for their successful adoption.

## Introduction and background

The term robotic process automation (RPA) refers to the application of technology to automate business operations under the guidance of structured inputs and business logic. Software robotics is another term for it. An organization can create a robot or software to record and interpret data for data manipulation, system communication, transaction processing, and response triggering by employing RPA technologies. There are various approaches to implementing RPA in the healthcare sector [1]. In healthcare, RPA involves the utilization of software robots to mimic human action, the time-consuming and repetitive tasks in healthcare operations such as managing medical records, scheduling patients, processing claims, and onboarding patients and employees. RPA bots automate manual processes, allowing employees to dedicate more time to decision-making [2].

Globally, the healthcare sector faces many challenges, including disparities in health outcomes, inadequate healthcare coverage, and fragmented information systems. However, there is a lot of potential to solve these problems using the combination of RPA and artificial intelligence (AI). Global healthcare systems can advance toward universal healthcare coverage and better health outcomes by utilizing these technologies to increase administrative effectiveness, improve diagnostic accuracy, and improve access to high-quality healthcare.

The use of AI with RPA in the healthcare sector has significantly increased in recent years. Healthcare activities like patient scheduling, registration, electronic health records, administrative tasks, admission and discharge procedures, robotic-assisted surgery, claims processing, and much more can be effectively handled by RPA tools and AI technology. Reducing manual labor and improving patient care are all possible outcomes of automation [2]. Administrators and medical professionals benefit greatly from using RPA, which enables them to complete jobs more efficiently and at a higher capacity. The combination of RPA with AI forms the next level of automation, as these technologies allow systems to learn, adapt, and optimize procedures independently. AI can assign jobs, which RPA then completes. Automation solutions may play a bigger role in the future, for instance, in the monitoring of individuals who require care [3].

AI's main area of importance in healthcare includes RPA, which replicates human workers’ back-office operations, like data extraction, form filling, and file transferring, using software; Natural Language Processing (NLP) is an area of AI that focuses on the analysis and modification of textual or spoken data produced by humans; these technologies enhance both clinical decision-making and administrative efficiency, complementing the use of RPA [4,5]. NLP is another essential component of AI. NLP allows computers to interpret and process human language, which is particularly useful for extracting information from clinical notes, patient feedback, and medical literature. By analyzing textual data, NLP enhances the decision-making process, making it easier to classify medical conditions or summarize patient histories [6]. Recognized for its ability to explore, examine, and understand large quantities of patient data, NLP is increasingly incorporated into the healthcare sector. Utilizing sophisticated medical algorithms, NLP technology can uncover valuable insights from clinical notes, effectively transforming unstructured data and leading to improved patient outcomes [6].

Machine learning involves the creation and application of systems capable of learning and adapting from data patterns without explicit instructions. AI employs machine learning methods such as XGBoost, Support Vector Machines, and Artificial Neural Networks to analyze complex data and identify patterns that traditional statistical methods might miss [7]. XGBoost is a powerful tool for making accurate predictions by learning from the errors of previous models [7]. Support Vector Machines are classification algorithms that use supervised learning to separate data into two groups by identifying the hyperplane that maximizes the margin between them, ensuring optimal separation and accurate classification [7,8]. Artificial Neural Networks consist of an input layer, one or more hidden layers, and an output layer, with each neuron in one layer connected to all neurons in the preceding and following layers, enabling complex data processing and pattern recognition [7,8]. Supervised machine learning techniques are widely used in the healthcare industry for image detection, hospital outcome identification, and disease prediction [8].

The World Health Organization (WHO) advocates for the evidence-based integration of AI in healthcare. The primary objective is to ensure that AI innovations positively impact global health through safe, ethical, and equitable means, supported by effective governance and regulation. The WHO acknowledges the vast potential of AI to transform healthcare and address critical issues like workforce shortages and limited resources [9]. AI in healthcare can be categorized into four main groups: expressive, analytical, predictive, and prescriptive applications. AI can help address the shortage of professional healthcare experts by diagnosing common health problems, saving time, decreasing the workload of expert health professionals, and reducing treatment costs in India [10].

The COVID-19 pandemic has significantly increased demand for essential medical supplies and equipment as well as modern RAP and AI applications. The workload of frontline workers has been significantly reduced by the intelligent robot systems' potential to assist in diagnosis, risk assessment, telehealth care, monitoring, disinfection, supply chain and delivery, service automation, and faster rates of pharmaceutical development, research, and various other tasks during COVID-19 pandemic [11]. Additionally, AI enhances the healthcare system by improving diagnosis and treatment, boosting patient engagement and adherence, and streamlining administrative tasks [4]. AI not only reduces the workload for doctors, nurses, and other healthcare professionals but also saves significant time. Therefore, embracing digital solutions for the prevention, diagnosis, and treatment of various ailments is a prudent approach for India to achieve the goal of health for all.

The main challenge is to extend these technologies to smaller towns and rural areas to make them accessible to a larger portion of the country's population. Furthermore, there is a significant number of empty positions for medical professionals at the grassroots level, and it is a challenge to find individuals who are trained and skilled in operating and maintaining robotics and AI technology [12]. The utilization of robotics in healthcare leads to significant legal issues as well. Similar to any other computer, the surgical robot can also be susceptible to virus threats, which could result in it not following the surgeon's command, potentially creating a dangerous scenario [4]. This review aims to explore how combining AI with RPA is transforming healthcare. It will highlight these technologies' benefits, challenges, and healthcare implementation. The goal is to provide insights to the healthcare industry to improve efficiency, accuracy, and patient care.

## Review

Methodology

This review explores the increasing trends of AI with RPA in healthcare, i.e., AI in healthcare, RPA in healthcare, digital health service, appointment scheduling, robotic-assisted surgery, claim management, medical billing, patient onboarding, patient registration, and record management, simplifying the procedure and enabling patients in the delivery of quality healthcare services. We search through databases such as Google Scholar and PubMed (Medline) along with government websites like the Ministry of Health and Family Welfare, the Ayushman Bharat Digital Mission, the National Health Portal of India, and the Ministry of Electronics and Information Technology. We found important articles through full texts from the past 10 years. We also checked the references in the bibliography of these articles. Our review includes studies from 2012 to 2023. We removed duplicates, abstracts, articles not in English, unpublished works, and references irrelevant to the objective and scope of our topic.

Artificial intelligence and robotic process automation

Every healthcare organization aims to offer outstanding healthcare to achieve positive patient outcomes and enhance patient experiences. Utilizing RPA with AI in healthcare can meet goals and build trust in patients about their treatment plans and decisions to access quality healthcare [13]. RPA is considered a technological tool with characteristics focused on humans in the digital transformation toolbox. Software robots can be used to take over repetitive and logical tasks that would typically require human involvement. These automated robots can efficiently carry out and organize tasks on their own, ensuring accuracy and transparency, ultimately leading to reduced expenses when compared to conventional automation techniques [14].

The combination of RPA with AI makes them more valuable and powerful, benefiting healthcare even more. The integration of AI with RPA offers in-depth analysis, giving healthcare professionals useful data to enhance decision-making and patient care coordination [15]. AI and RAP span appointment scheduling, robotic-assisted surgery, administrative data entry, telehealth monitoring, claim management, EHR, medical billing, patient and staff boarding, and other possible uses in the healthcare sector. We illustrate the significant contribution of AI and RAP in healthcare during the COVID-19 pandemic increase in demand for essential drug supply and equipment.

Application, advantage, and use of artificial intelligence with robotic process automation in healthcare

**Figure 1:**
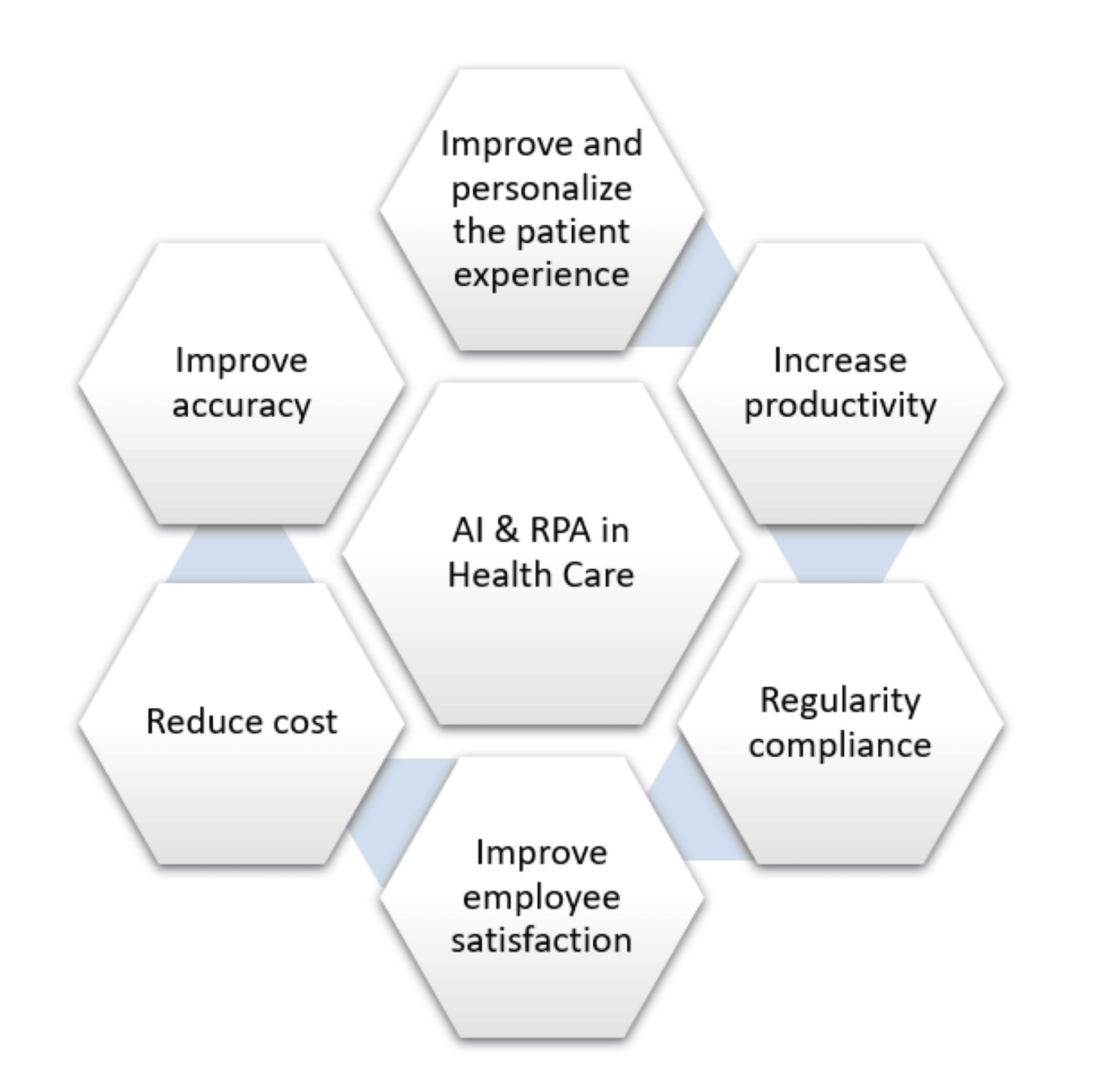
Application of AI and RPA in healthcare AI, artificial intelligence; RPA, robotic process automation

The combination of AI with RPA in healthcare leads to smart automation, enhancing healthcare processes. Although RPA with AI are viewed as distinct fields, they work well together. According to this review, most research on AI and RPA in healthcare focuses primarily on benefits, uses, and applications, such as appointment scheduling, administrative data entry, telehealth monitoring, EHR, robotic-assisted surgery, medical billing, patient and staff onboarding, and many more [16]. The following section highlights some of the major practical applications of AI with RPA in various healthcare sectors. Figure 1 shows the applications of AI with RAP in healthcare [17].

Reduce Cost

RPA and AI reduce healthcare costs by automating administrative tasks, minimizing errors, and streamlining operations. These technologies optimize resource allocation, enhance efficiency in billing and claims processing, and reduce the need for manual labor. By improving diagnostics and patient management, they also help prevent costly medical errors and unnecessary procedures, ultimately lowering overall expenses. Additionally, implementing RPA enhances efficiency in workforce utilization, time management, and resource allocation. RPA performs tasks more quickly, precisely, and with less human involvement, leading to more sustainable healthcare operations amidst rising costs. The financial benefits of these efficiencies positively impact patient care by improving the organization's overall effectiveness [18].

Regularity Compliance

RPA helps healthcare organizations stay compliant with various regulations by automating the creation and submission of reports. This automation enables healthcare providers to achieve regulatory compliance more efficiently and accurately, reducing the likelihood of errors. Additionally, RPA's role in data management and reporting supports process optimization and adherence to regulations concerning patient data. These use cases underscore the importance of implementing RPA in the healthcare industry for accurate recordkeeping and minimizing mistakes [17,19,20].

Increase Productivity

RPA helps healthcare organizations stay compliant with various regulations by automating the creation and submission of reports. This automation enables healthcare providers to achieve regulatory compliance more efficiently and accurately, reducing the likelihood of errors. Additionally, RPA's role in data management and reporting supports process optimization and adherence to regulations concerning patient data. These use cases underscore the importance of implementing RPA in the healthcare industry for accurate recordkeeping and minimizing mistakes [17,19,20].

Improve and Personalize the Patient Experience

RPA and AI enhance and personalize the patient experience by offering tailored treatments, efficient communication through chatbots, and quick access to medical information and support. Healthcare professionals are utilizing RPA to enhance patient satisfaction by automating processes such as scheduling appointments, handling medical billing, and processing claims to minimize wait times and increase overall health facilities [19]. Decreasing staff participation in repetitive activities gives them more time for patient interactions, enables more individualized and comprehensive care, simplifies patient enrolment, and enhances access to the complete patient history for improved decision-making [17].

Improve Employee Satisfaction

RPA mitigates burnout and enhances overall job contentment by alleviating employees from repetitive duties. Employees who are empowered help create a positive work atmosphere, leading to improved patient experiences [18].

Improve Accuracy

**Figure 2:**
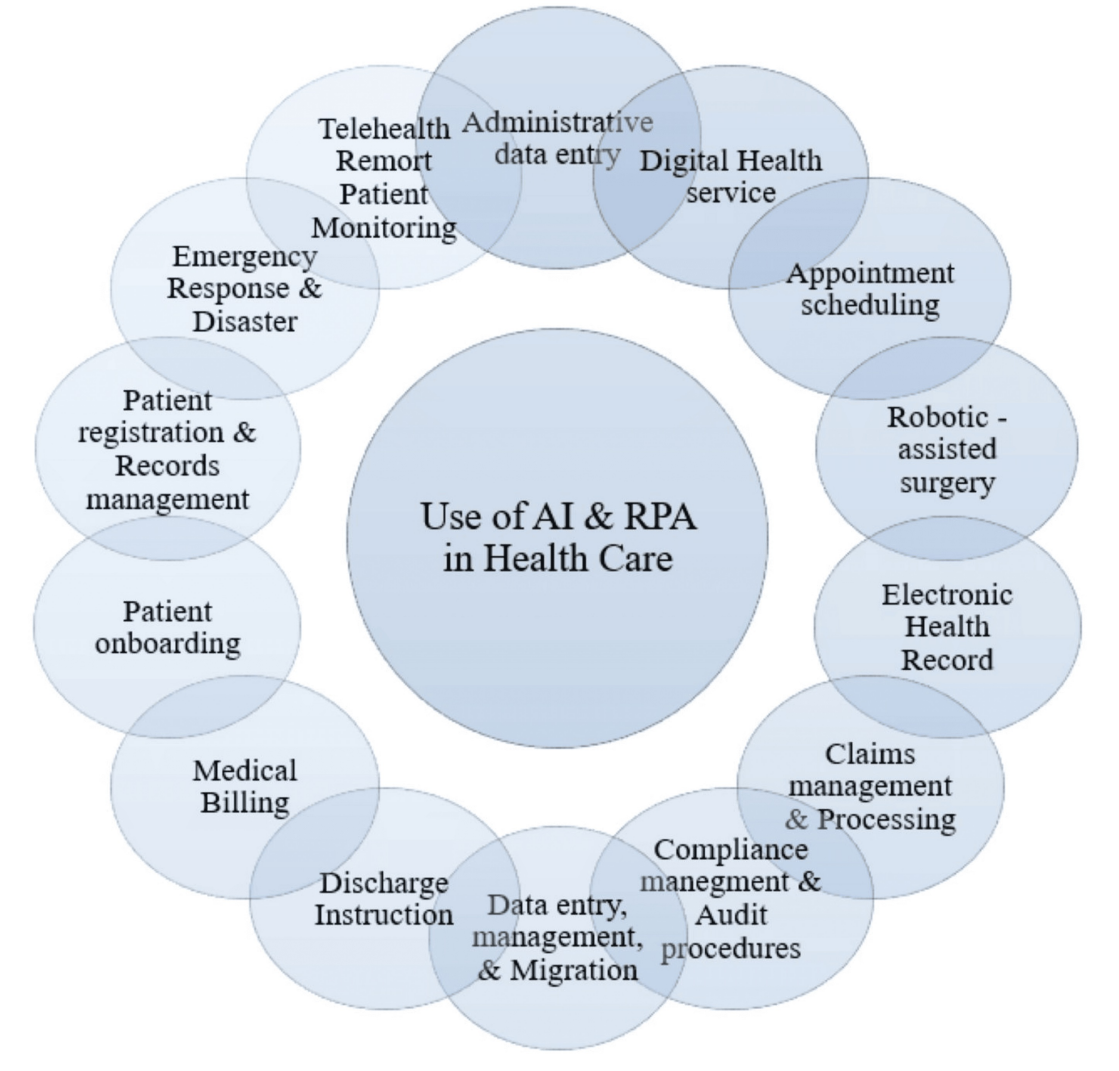
Use of AI and RPA in healthcare AI, artificial intelligence; RPA, robotic process automation

By utilizing RPA, healthcare tasks are completed with fewer mistakes, eliminating the risks associated with manual data management. This can lead to improved precision and uniformity in different procedures, leading to more informed decision-making and decreased risks linked to mistakes made by humans [18]. The following section highlights some uses of AI and RPA in various healthcare sectors. Figure 2 shows the use of AI and RPA in healthcare [2].

Medical Billing

The billing process significantly impacts patient experience, and RPA enhances patient satisfaction by expediting claims processing and ensuring timely, accurate billing statements. Automated systems minimize billing errors and disputes, resulting in a smoother, more transparent billing process [21]. Billing and claims interactions and tasks between healthcare providers and insurance companies are usually repetitive. However, with RPA bots, healthcare organizations can automate processes for managing various tasks like follow-ups and first-line inquiries [17,19].

Electronic Health Records and Patient Registration

EHR encompass the electronic representation, storage, and organization of health information for individuals. Typically, service providers store these records individually, maintaining control and perhaps restricting data sharing with other healthcare partners [5,22]. Instead of humans managing health records, RPA bots can track records received from multiple sources every hour, downloading PDFs and other medical details from various documents using optical character recognition. EHR have the potential to transform the healthcare industry [2]. Using RPA bots to automate patient registration can accelerate the entire registration process, including account setup, history verification, element processing, and bill management.

Patient Onboarding 

RPA assists healthcare institutions in effortlessly gathering patient details such as medical records, insurance data, and demographics, enabling employees to establish or modify their EHR and electronic medical records [17]. RPA can simplify the patient onboarding procedure in healthcare by automating tasks like data entry, verification, paperwork, payment processing, and scheduling. RPA implementation in healthcare organizations enhances onboarding, boosts patient satisfaction, and increases efficiency, accuracy, and cost-effectiveness [23].

Robotic-Assisted Surgery

Robotic-assisted surgery, a significant application of RPA in healthcare, enhances surgical precision, reduces human error, and improves patient outcomes while minimizing recovery times and hospital stays. The idea of using robotics for surgery was first envisioned in 1967, but it remained a vision for approximately three decades until research organizations established by the US Defense Department created the first surgical robot capable of performing various tasks [24]. Physicians have the option of utilizing robots to assist in performing intricate surgeries with minimal incisions. Surgical robotics is the most popular application of robotics in the field of healthcare and medicine. They allow surgeons to make more accurate cuts and have supported the creation of new minimally invasive techniques [25].

Telehealth and Remote Patient Monitoring

Telehealth and remote patient monitoring, utilizing AI and RPA, enable continuous, real-time monitoring of patient's health information. This integration allows for timely interventions, enhances patient care, reduces hospital visits, and maximizes resource usage by automating regular tasks and data analysis [4,26].

Appointment Scheduling

By utilizing RPA, healthcare institutions can improve the efficiency of appointment scheduling and eliminate time-consuming manual tasks. This technology can assess and utilize incoming data, such as patient symptoms and doctor availability, to simplify the appointment scheduling process, which enhances patient admission and discharge procedures [17]. Automating patient appointment scheduling can increase patient satisfaction and improve the experience of physicians, nurses, and staff. RPA helps in more efficient appointment scheduling, which results in better patient care [27]. RPA bots can learn to schedule patient appointments based on a set workflow that considers factors such as doctor availability, location, and diagnosis. Additionally, these bots provide assistance and support across multiple channels [2].

Administrative Data Entry

RPA can automate various administrative tasks within the healthcare sector, such as data input, appointment scheduling, and insurance claims processing. By automating tasks like collecting patient information, verifying insurance eligibility, and scheduling appointments, RPA significantly reduces the workload of healthcare workers. This not only improves productivity but also saves time and effort for healthcare staff [28]. Additionally, administrative data entry, a crucial task in healthcare, is often prone to errors when performed manually. RPA can mitigate these issues by accurately gathering data from multiple sources, organizing it, and entering it into a database [17].

*Claim Management and Processing* 

Automation and RPA tools play a vital role in managing healthcare insurance claims by automatically populating insurance fields, using standard fields as appropriate, creating claims documents, and storing information in centralized systems [2]. Healthcare organizations handle a large volume of claims, which are often repetitive and time-consuming to process. RPA allows these organizations to automate the process using rules-based decision-making, thereby reducing errors and ensuring compliance with regulations [17,19].

Reducing the Risk of Human Error and Decision-Making

Implementing AI and RPA reduces human errors in critical healthcare tasks by eliminating the pitfalls of manual data handling. This can result in enhanced accuracy and consistency across various processes, contributing to better decision-making and reducing the risks associated with human errors [18].

Digital Health Service

The integration of digital health services with RPA and AI is transforming the delivery of healthcare by rearranging processes for better care and greater efficiency. This integration reduces administrative burdens, minimizes errors, and gives more accurate and timely medical information so that better care outputs can be delivered with efficient healthcare [5]. Government digital health services utilize AI, machine learning, and RPA in healthcare.

Opportunities and advantages of robotic process automation in private healthcare

Organizations are increasingly using digitalization to handle the increasing complexity of their processes. In the private healthcare sector, there is a strong focus on improving performance as the industry continues to evolve. As a result, automation technologies, such as RPA, are gaining popularity as effective solutions to meet this need [29-31]. The automation of workflow processes is now a common approach to tackling operational obstacles [31]. This review evaluates how RPA can add value to the private healthcare industry by examining its functionalities and applying Walter et al.'s function-oriented value analysis to identify its potential benefits.

Limitations, challenges, and future scope of artificial intelligence with robotic process automation in healthcare

RPA offers significant benefits in healthcare, such as increased efficiency and reduced errors. However, several challenges accompany its implementation. One major limitation is the high initial cost and complexity of integrating RPA systems into existing healthcare IT infrastructure. This integration can be resource-intensive and requires specialized knowledge, which may not be readily available in all healthcare institutions. Additionally, RPA's effectiveness is heavily dependent on the quality of existing data and systems; poorly structured or inaccurate data can lead to suboptimal automation outcomes [25].

The main challenge is accessing the distant areas of the country with limited infrastructure and the absence of advanced technologies. The exorbitant cost is a significant obstacle to providing AI and robotics in healthcare to underserved communities. Moreover, errors and mechanical failures may occur as a result of inadequate maintenance arrangements, leading to potentially deadly outcomes [4].

**Figure 3:**
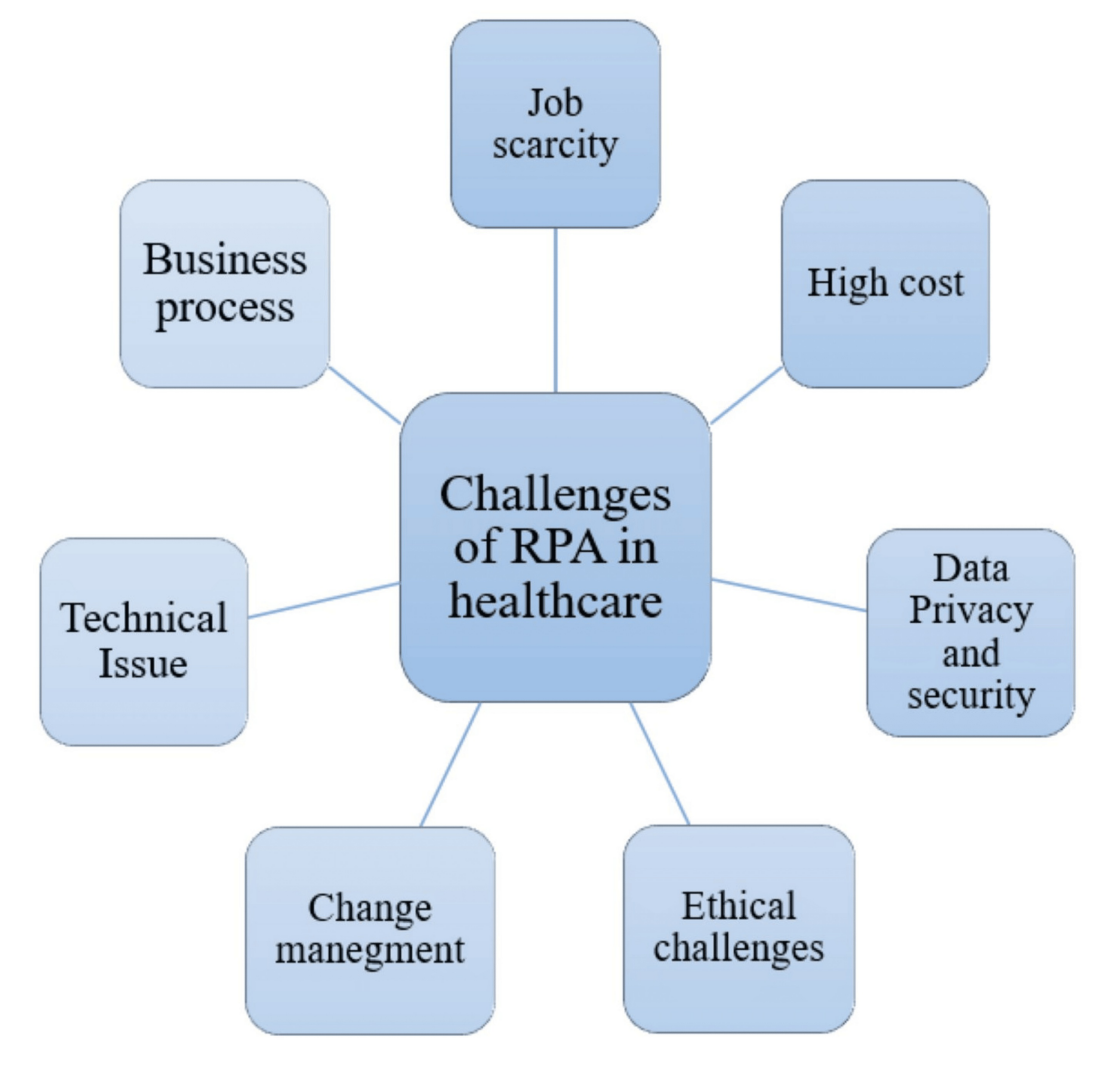
Challenges of RPA in healthcare RPA, robotic process automation

Incorporating RPA into the healthcare field brings forth promising prospects and unique obstacles. Managing the intricacies of the healthcare sector requires consideration even though there is a chance to enhance efficiency and productivity through processes [29]. The healthcare service desk agent faces a challenging task and workflow overhaul. Furthermore, when the number of patients is high, managing several tasks simultaneously and providing satisfactory solutions becomes even more challenging, impacting overall patient satisfaction [2]. Furthermore, the integration of RPA in healthcare involves addressing issues such as job displacement, high costs, data privacy and security concerns, ethical challenges, business processes, job scarcity, change management, and technical issues [32]. Figure 3 shows the challenges of RPA in healthcare [32].

In the future, using RPA in healthcare shows a lot of promise for automating tasks that need a lot of work. Robotics automation can be used in healthcare processes like entering data integrating systems and making decisions, in healthcare facilities and homecare settings. The combination of RPA tools and AI technologies has the potential to handle tasks such as scheduling patients, registering them, managing health records, handling admissions and discharges, and processing claims efficiently. By using automation, healthcare operations can improve efficiency, reduce work, and enhance the quality of care [2].

The healthcare industry is rapidly evolving with modern technology, such as machine learning, AI, and robotics. These innovations transform the sector by creating intelligent machines that can work and respond like humans. While still in its early stages, the future of AI and robotics in healthcare is promising, with the potential for widespread acceptance and success [4]. The areas in healthcare where AI and robotics can quickly adapt are drug discovery, care for elderly people, boost in clinical trials, consultation in digital mode, AI in diagnosis, consultation in digital mode, AI in nanotechnology research, and prediction of an epidemic outbreak [4]. Table 1 shows a summary of the included studies.

**Table 1:** List of included studies in the review AI, artificial intelligence; RPA, robotic process automation

Author	Year	Type of article	Findings
Chatterjee S et al. [10]	2021	Review article	Indian healthcare sector embracing AI for enhanced services, encountering security and regulatory challenges. Positive views on AI attracting local and global companies. Balanced policy is essential for promoting innovation and averting misuse. Guidelines proposed for effective AI implementation in Indian healthcare.
Deo N et al. [4]	2023	Review article	Healthcare in India embraces robots and AI for drug discovery, diagnostics, surgeries, and elder care. Challenges include access, costs, maintenance, and ethics, requiring government support and partnerships for success.
Sarker S et al. [11]	2021	Review article	AI and robotics sped up COVID-19 vaccine research with data analysis tools and RPA is a platform for distribution. They set a model for disaster responses, showing professionals' dedication.
Shaheen MY et al. [1]	2021	Review article	RPA uses technology to automate business processes in healthcare, including scheduling appointments, reminding patients of instructions, streamlining claims, managing workflows, and speeding up account settlements.
Ambar M [15]	2024	Website	Healthcare organizations aim to offer top-notch healthcare for positive patient outcomes. By using RPA and AI, staff can focus on patient care while automation streamlines processes, leading to improved patient experiences.
Huang et al. [14]	2024	Case study	This study confirms a practical example of lean digital transformation in healthcare organizations. It allows for reallocating human resources to more valuable and creative tasks, while also helping hospitals offer more comprehensive and patient-focused services.
Ivancic L et al. [13]	2019	Article within a book	The review aims to examine how the academic community defines RPA and its investigation in the literature regarding its state, trends, and application. It also compares RPA to business process management through a systematic literature review.
Kitsantas T et al. [16]	2024	Original article	The review explores RPA-AI integration benefits in revolutionizing business processes. Findings highlight automation opportunities, address technical challenges, evaluate processes, and innovate techniques.
Kaur J [33]	2023	Review article	RPA is an innovative technology that eliminates repetitive tasks, undergoing research for academic study. It is not traditional robotics but a modern, rapidly growing field known as RPA. Its application in healthcare is highlighted in the study.
M. Ratia et al. [34]	2018	Article within a book	Organizations are adopting digitalization to handle diverse processes. The private healthcare sector aims to enhance performance as the industry evolves. RPA is increasingly popular in automating workflow processes.

## Conclusions

Integrating AI and RPA in healthcare presents a transformative opportunity to improve efficiency, accuracy, and patient outcomes. These technologies offer significant benefits, from automating administrative tasks to enhancing decision-making and patient care. However, challenges such as data privacy, implementation costs, and regulatory issues must be addressed to realize their potential fully. As AI and RPA technologies continue to evolve, they are expected to play an increasingly vital role in healthcare, driving innovation, and improving the quality of care. The future holds great promise for AI and RPA, with advancements in predictive analytics, precision medicine, and patient engagement, ultimately leading to a more efficient, effective, patient-centered healthcare system.
